# Proteomic cellular signatures of kinase inhibitor-induced cardiotoxicity

**DOI:** 10.1038/s41597-021-01114-3

**Published:** 2022-01-20

**Authors:** Yuguang Xiong, Tong Liu, Tong Chen, Jens Hansen, Bin Hu, Yibang Chen, Gomathi Jayaraman, Stephan Schürer, Dusica Vidovic, Joseph Goldfarb, Eric A. Sobie, Marc R. Birtwistle, Ravi Iyengar, Hong Li, Evren U. Azeloglu

**Affiliations:** 1 Department of Pharmacological Sciences and Institute for Systems Biomedicine, Icahn School of Medicine at Mount Sinai, New York, NY 10029 USA; 2 Center for Advanced Proteomics Research and Department of Microbiology, Biochemistry and Molecular Genetics, Rutgers University - New Jersey Medical School, Newark, NJ 07103 USA; 3 Department of Molecular and Cellular Pharmacology, Miller School of Medicine, University of Miami, Miami, FL 33136 USA; 4 Center for Computational Science, University of Miami, Miami, FL 33136 USA; 5 Department of Chemical and Biomolecular Engineering, Clemson University, Clemson, SC 29634 USA; 6 Department of Medicine, Division of Nephrology, Icahn School of Medicine at Mount Sinai, New York, NY 10029 USA

**Keywords:** Proteomics, Drug development

## Abstract

Drug Toxicity Signature Generation Center (DToxS) at the Icahn School of Medicine at Mount Sinai is one of the centers for the NIH Library of Integrated Network-Based Cellular Signatures (LINCS) program. Its key aim is to generate proteomic and transcriptomic signatures that can predict cardiotoxic adverse effects of kinase inhibitors approved by the Food and Drug Administration. Towards this goal, high throughput shotgun proteomics experiments (308 cell line/drug combinations +64 control lysates) have been conducted. Using computational network analyses, these proteomic data can be integrated with transcriptomic signatures, generated in tandem, to identify cellular signatures of cardiotoxicity that may predict kinase inhibitor-induced toxicity and enable possible mitigation. Both raw and processed proteomics data have passed several quality control steps and been made publicly available on the PRIDE database. This broad protein kinase inhibitor-stimulated human cardiomyocyte proteomic data and signature set is valuable for prediction of drug toxicities.

## Abstract (summary)

**Table:** 

Measurement(s)	protein expression profiling
Technology Type(s)	mass spectrometry assay
Factor Type(s)	Individual proteome • Kinase inhibitors
Sample Characteristic - Organism	Homo sapiens

Machine-accessible metadata file describing the reported data: 10.6084/m9.figshare.16990033

## Background & Summary

Protein kinase inhibitors (KIs) belong to a class of targeted therapeutics that are being increasingly used in treatment of various cancers^1^. Their use and development have been accelerated in recent years as they could target tumors more effectively than most other chemotherapeutics, and their mechanisms of actions are well defined. In many cases, however, their intended- or off-target kinases serve key biological roles, which when blocked, lead to severe adverse effects^2^. One of the major adverse effects that lead to discontinuation of treatment with KIs is cardiotoxicity^3^. As a part of the NIH Library of Integrated Cellular Signatures (LINCS) program^4^, the main goal of the Mount Sinai Drug Toxicity Signature Generation Center (DToxS) is to better understand mechanisms of KI-associated cardiotoxicity by constructing cellular signatures of drug effects. Since different omics assays show varied sensitivities^5^ and offer complementary molecular information on the cellular phenotypic state^6^, in order to develop a comprehensive understanding of the cellular responses to KIs, we use network analyses^7^ to combine differential expression of genes and gene products in human cardiomyocytes treated by FDA approved KIs, analyzed using both transcriptomic and proteomic methods. In this dataset, we present the proteomic portion of the effects of KIs on human cardiomyocytes. Kinase inhibitors presented here are grouped and color-coded according to their primary target profile (see Table 1 for this and additional drug metadata).

**Table 1:** Drug metadata.

Drug	Acronym	Drug Form	Purity	Concentration (μM)	Reference
Afatinib	AFA		98.8	0.05	^27,28^
Axitinib	AXI		98.0	0.20	^29,30^
Bosutinib	BOS		98.0	0.10	^31^
Cabozantinib	CAB		99.1	2.00	^32^
Dabrafenib	DAB		99.6	2.50	^33,34^
Dasatinib	DAS		98.4	0.10	^35^
Erlotinib	ERL		99.3	3.00	^36,37^
Gefitinib	GEF		99.3	1.00	^38^
Imatinib	IMA		99.5	5.00	^39^
Lapatinib	LAP		99.0	2.00	^40^
Nilotinib	NIL		99.8	3.00	^41,42^
Pazopanib	PAZ		96.1	10.0	^43^
Ponatinib	PON		97.3	0.10	^44^
Regorafenib	REG		99.6	1.00	^45^
Ruxolitinib	RUX		99.4	1.00	^46,47^
Sorafenib	SOR	Tosylate	99.7	0.50	^46,47^
Sunitinib	SUN	Malate	97.1	1.00	^48,49^
Trametinib	TRA		99.2	0.10	^50^
Tofacitinib	TOF	Citrate	99.9	1.00	^50^
Vandetanib	VAN		99.7	0.33	^51^
Vemurafenib	VEM		98.1	2.00	^52^

Purity was verified for each lot number. The concentration used for each drug is equivalent to the clinically-observed median peak plasma concentration as per the cited reference. Drugs are coded into different shades of a given color based on their primary target: purple (EGFR), orange (BCR-ABL), gray (VEGFR), red (JAK1/2), pink (MEK1/2), blue (multi-kinase).

Quantitative proteomics technologies have evolved to become increasingly effective at identifying differentially expressed proteins among diverse experimental conditions. Currently, two broad strategies are widely used for large-scale quantitative proteomics studies: stable isotope label-based^8,9^ and label-free quantification (LFQ) strategies^10,11^. The label-based methods provide fewer experimental variations and better protein quantitation precision^12,13^; however, the cost for label-based proteomics reagents is prohibitively expensive for analyzing hundreds of samples. LFQ methods, on the other hand, do not involve expensive labeling reagents; thus, they can be used for clinically relevant studies of hundreds and thousands of samples if proper controls and quality guidelines are followed. LFQ methods have more comprehensive dynamic ranges and more flexibility in study design^14–16^. However, while LFQ sample throughput can be increased through this approach, it should be noted that this approach can also lead to a smaller number of identified proteins, due to limited peptide fractionation.

**Fig. 1:**
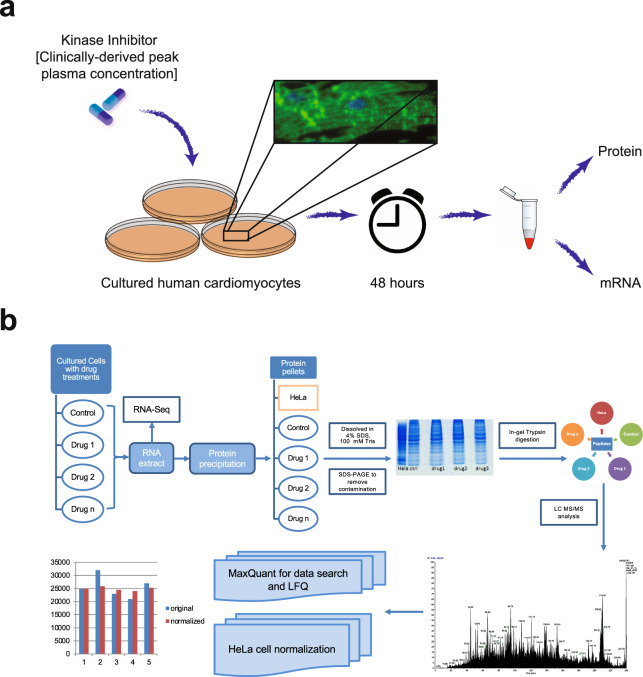
LFQ proteomics workflow for the DToxS dataset. (**a**) Drug and cell treatment design and experimental workflow. (**b**) After drug treatment, RNA was extracted from cell pellets, and the remaining proteins were recovered via protein precipitation. The protein amount for each sample was carefully estimated from its SDS-PAGE staining intensity as measured relative to a HeLa cell lysate standard. Two micrograms of protein from each sample were analyzed by LC-MS/MS, and the resulting proteins were quantified via LFQ approach using MaxQuant.

**Fig. 2:**
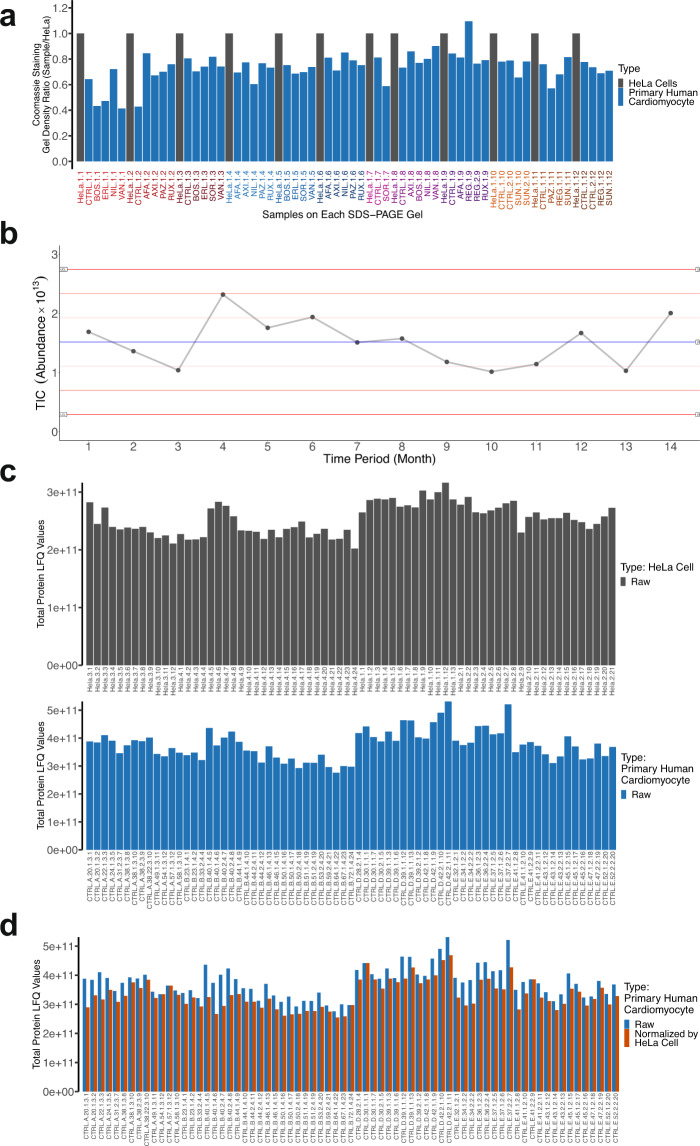
MS/MS quality control for the DToxS dataset. A series of quality control steps have been implemented to obtain high quality data for this dataset. (**a**) A gel-based method was developed for protein estimation. In each SDS-PAGE gel, 25 µg of HeLa cell protein extract was run as a reference to estimate the total protein amount of each sample (blue verticle lines). Based on the density of the CBB stain of each lane in relation to the HeLa stain density, the protein amount can be calculated (see examples in Online-only Table 1). After the tryptic digestions, the resulting peptides were diluted into 0.5 µg/ml for LC-MS/MS analysis (orange verticle lines), based on the estimated protein amount in each sample. (**b**) Levey-Jennings performance chart showing instrument stability. Two hundred nanograms of commercial HeLa protein digest was analyzed regulary during the time period of the DToxS LINCS data acquisition. Total ion current (TIC) from each month is shown. Red lines demarcate one, two and three times the standard deviation; LCL/UCL = lower/upper control limit. (**c**) Deep proteome coverage indicates good instrument sensitivity. Total protein groups identified from each cell line in this study are shown, with over ~5,000 unique proteins identified per cell line. (**d**) The HeLa protein digests were used for LFQ normalization. Based on the MS1 LFQ counts of the HeLa cells from each gel (top panel), a normalization factor was calculated and applied to the MS1 counts of the samples. After the HeLa cell normalization, the LFQ variation was more stable.

In this DToxS dataset, we have chosen the LFQ method in order to economically compare the proteomics signatures from over 300 samples (Supplementary Table 1) that have been collected over an extended period of time, some with limited protein yields. The transcriptomes of these samples have been previously assessed using 3′ digital gene expression RNA sequencing (RNAseq)^17,18^. To ensure both proteomic and transcriptomic data can be compared from the same drug-treated cells, we have optimized a method to extract proteins from the samples after RNA extraction at the Center for Advanced Proteomics Research (CAPR, http://njms.rutgers.edu/proweb/) at Rutgers University - New Jersey Medical School (Fig. 1). All the sample preparation, analysis procedures and data have gone through careful quality control steps to ensure reproducibility (Fig. 2); standard operating procedures and data descriptors are publicly available in the DToxS website (www.dtoxs.org) and both raw and MaxQuant-analyzed data are publicly available via the PRIDE database (PXD014791) as well as the LINCS Project data portal (http://lincsportal.ccs.miami.edu/dcic-portal/). We think that this large KI-induced proteomics dataset will be a valuable resource for cancer, cardiology and drug development communities.

## Methods

### Cell culture, drug treatments and RNA extraction

Detailed materials and methods for all the experiments in this study can be accessed as version- and quality-controlled standard operating procedures on DToxS.org. Briefly, four commercially available cell lines of primary adult human cardiomyocytes were purchased from PromoCell GmbH (Cat #: C12810; Heidelberg, Germany), expanded and differentiated under serum-free conditions for 28 days per manufacturer’s instructions. The four cell lines used in this study (Lot #: 3042901.2, 4031101.3, 2082801.2, 2120301.2) were isolated from two Caucasian male and two Caucasian female subjects, aged 54, 62, 61, 56, respectively (see cell lines PMC-A, B, D, E in Table 2). Although these cell lines were originally derived from the heart and have many cardiomyocyte-like properties, they are not excitable or contractile; hence, we refer to them as cardiomyocyte-like throughout the dataset. All four cell lines were subjected to rigorous standardized quality control metrics that included regular testing for mycoplasma contamination and confirmation of cardiac origin through RT-PCR screening of key cardiac genes (ACTN2, TNNT2, KCHN2, MEF2C, NKX2–5 and GATA4), immunofluorescent validation of cardiac-specific actinin crossfiber formation, and measurement of calcium activity. Details of extended cellular metadata for each line can also be found at DToxS.org. Briefly, cells were subcultured at 37 °C under 5% CO_2_ with the manufacturer-supplied and serum-supplemented growth media (Cat #: C22270, C39275) up until passage four. Once they reached 100% confluence, cells were trypsinized, counted, and replated in serum-free growth media (Cat #: C22270), at a concentration of 40,000 cells/cm^2^ in 60 mm tissue culture dishes. Cells were then differentiated under serum-free conditions for 28 days, whereby 50% of the media was replenished every other day. All experiments were performed on sixth or earlier passage cells. After 28 days of differentiation, cells were treated with individual KIs for 48 hours at a concentration equivalent to clinically-observed median peak plasma concentration. Forty-eight hours was chosen to mimic the *in situ* chronic conditions and to detect consistent, non-transient, proteomic changes. We note that no extensive cell death or apoptosis were observed. Before and after treatment cell counts and bright field images were obtained to quantify changes in cell numbers. After 48 hours of KI treatment, cells were lysed on ice using TRIzol (Thermo Fisher, Cat #: 15596026) for five minutes, scraped off the dish, and mixed. TRIzol lysates were mixed with chloroform according to manufacturer’s instructions and the RNA-protein fractions were separated. From these organic partitions, RNA samples were processed, sequenced and analyzed as previously reported^18^. Results of the transcriptomic signatures are reported elsewhere^17^.

**Table 2:** Cellular metadata.

Cell Name	Vendor	Cat No	Lot No	Race	Sex	Age	Disease
PMC-A	PromoCell	C-12810	3042901.2	Caucasian	Male	54	None
PMC-B	PromoCell	C-12810	4031101.3	Caucasian	Female	62	None
PMC-D	PromoCell	C-12810	2082801.2	Caucasian	Female	61	None
PMC-E	PromoCell	C-12810	2120301.2	Caucasian	Male	56	None

### Protein extraction

After isolating RNA, the remaining TRIzol solutions were processed for protein extractions (Fig. 1a). Each TRIzol extract were first separated into two 1.5 mL Eppendorf tubes; each tube contained ~600 µL of the TRIzol chloroform and methanol solvent mixture. Then to each tube, 900 µL of pre-chilled cold acetone was added and mixed well. Five hundred µL of the mixture was transferred from each tube to a third new tube, and 500 µL pre-chilled −20 °C acetone was added to each of the three tubes and mixed well. All three tubes were put on ice for at least 12 hours inside a cold room, and centrifuged at 16,000 rpm (~23,469 g) for 30 minutes, in a Beckman Coulter Allegra 64 R centrifuge. After centrifugation, the supernatants were removed and 1 mL of pre-chilled acetone was added to each tube to wash the protein pellets. The protein pellets were sonicated for five bursts at level-3 on a Branson Ultrasonic cleaner, repeated once. The protein solutions were placed on ice for four hours and pelleted *via* centrifugation as described above. The wash procedure for each protein pellet was repeated twice for a total of three times. The final protein pellet was washed with 50 µL of pre-chilled acetone and centrifuged at 16,000 rpm for 10 minutes, and the remaining pellet was air dried at room temperature for 10 to 15 minutes. The protein pellet was then resolubilized with a vortex mixer, with 70 µL of 8 M urea (Fisher Cat #: U15500) in 50 mM ammonium bicarbonate (Sigma Cat#: 09830-500 G), and stored at −80 °C before proteomics analysis. See additional protocol details at https://martip03.u.hpc.mssm.edu/sop.php.

### Sample preparation for proteomics analysis

The protein solutions may contain TRIzol and other low mass contaminants from the RNA extraction buffer, which can confound accurate protein estimation via conventional biochemical assays. Consequently, we ran the SDS-PAGE gels to remove the low mass contaminants from the high mass proteins, and estimated the protein amounts via the stain intensities from the gels stained with the Coomassie brilliant blue (CBB) dye (Fig. 1b and Supplementary Fig. 1). In each gel, 25 µg of a HeLa cell extract produced at the CAPR (aliquots of the same preparation were used for all gels) was run in one lane as the normalization reference, along with the proteins isolated from the drug-treated cell lines. After CBB staining of the gels, the density of each sample lane was measured using the ImageJ software (NIH), and the protein amount for each drug-treated sample was calculated using the following equation: protein (µg) = HeLa protein 25 µg × (Sample density/HeLa density). The protein quantity information was later used to dilute the tryptic peptides into 0.5 µg protein equivalents per µL for the LC-MS/MS analysis (Online-only Table 1).

The in-gel digestions of proteins were performed following a procedure similar to the one described by the Mann lab^19^. In brief, the entire gel lane of each sample was excised into ~1 cm^2^ gel blocks, and washed four times with 10 mL each of a Wash Buffer containing 30% acetonitrile (ACN) and 70% of 100 mM NH_4_HCO_3_ to remove the contaminants from the RNA extraction buffer. Subsequently, one mL of 25 mM dithiothreitol (DTT) solution was added to the gel blocks for disulfide reduction at 55 °C for 30 minutes, and 1 mL of 50 mM iodoacetamide solution was then added for thiol alkylation at 37 °C in the dark for 30 minutes. After alkylation, the gel blocks were dehydrated with 9 mL of ACN to remove both DTT and iodoacetamide. For in-gel trypsin digestion, one mL of trypsin solution (5 µg/mL in 50 mM NH_4_HCO_3_) was added into each sample, and incubated at 37 °C for 16 hours. Resulting peptides were extracted, desalted with Pierce C_18_ spin columns (Thermo Scientific) based on the manufacturer’s protocol and concentrated in a Speed Vac prior to LC-MS/MS analysis.

### LC-MS/MS analysis

According to the gel-based protein amount estimates described above, the peptides from each sample were resuspended in Solvent A (2% ACN in 0.1% formic acid (FA)) at a final concentration of 0.5 µg/µL (see examples in Online-only Table 1). Two micrograms of peptides from each sample were subjected to LC-MS/MS analysis on a Q Exactive Mass Spectrometer coupled with an UltiMate 3000 RSLCnano Duo LC system (Thermo Scientific). The peptides were first loaded onto a trapping column (Acclaim PepMap 100 C_18_ trap column 75 µm × 2 cm, 3 µm, 100 Å) and then separated on an Acclaim PepMap C_18_ column (75 µm × 50 cm, 2 µm, 100 Å), using a 4-h binary gradient from 2–95% of Solvent B (85% ACN in 0.1% FA), at a flow rate of 250 nL/min. The eluted peptides were directly introduced into the MS system for data-dependent MS/MS analysis in the positive ion mode. The MS full scans were acquired in an m/z range of 400 to 1750 in the profile mode, with the AGC value specified at 3E6, and the injection time set at 100 msec. The resolution of the full MS scan was set to 140,000 at m/z 400. Following each full MS scan, the 15 most intense ions with charge states between 2^+^ to 5^+^ were selected within an isolation window of 2 m/z for the subsequent MS/MS analysis. The AGC of MS/MS analysis was set to 5E4 and the dynamic exclusion was 45 sec. The peptide ions were fragmented using higher energy collision dissociation at a NCE of 27. A total of 308 LC-MS/MS raw files were obtained for the proteins isolated across four different cell lines for 60 different types of drug treatment conditions. The reference HeLa digest from each SDS-PAGE gel was analyzed using the same method to normalize the LFQ intensities from the other cell digests. To quality control the LC-MS/MS system performance, we also ran a Pierce HeLa Protein Digest Standard (Pierce, Cat# 88329) on the instrument, prior to running each set of drug-treated samples.

### MaxQuant for protein identification and quantification

In order to evaluate the quality of the data and compare the quantitative proteomic signatures among the drug-treated samples across different cell lines, the entire 372 raw LC-MS/MS dataset (308 samples and 64 HeLa cell controls, ~825 GB of data) was submitted for database search using the Andromeda search engine on the MaxQuant platform (Version 1.6.0.13). The raw data files were loaded with “No fractions” option selected. Trypsin was selected as enzyme with two miss cleavages. Methionine oxidation (+15.9949 Da) and protein N-terminal acetylation (+42.0106 Da) were selected as various modifications and cysteine carbamidomethyl modification (+57.0215 Da) was set as a fixed modification. Initial search peptide mass tolerance was set to 20 ppm, and the main search peptide mass tolerance was set to 4.5 ppm. LFQ was selected for label-free quantification, with the minimal LFQ ratio count set at 2. The MS/MS spectra were searched against both UniProt human FASTA database (downloaded from https://www.uniprot.org/proteomes/UP000005640 with the last modification date of 10/22/2018, containing 73,101 human protein sequences) and the MaxQuant default contaminants FASTA database (containing 245 protein sequences). Match between runs was selected to maximize protein identification and quantitation with a match time window of 0.7 minutes. The protein false discovery rate (FDR) was estimated using the decoy databases containing reversed sequences of the original proteins. Proteins identified (Table 3) with both protein and peptide FDRs at or less than 1% were included in the final results for the subsequent analyses.

**Table 3:** Summary of MaxQuant analytics.

Group	PMC-A	PMC-B	PMC-D	PMC-E
Total Identified Protein Groups	4,288	4,635	4,881	5,227
Total Identified Peptides	1,552,043	3,176,801	1,980,185	3,141,618
Median LFQ Intensity	14,366,500	15,757,000	13,341,000	12,479,000

## Data Records

All the raw data described in this study have been uploaded to the PRIDE repository at the ProteomeXchange website (https://identifiers.org/pride.project:PXD014791) with the project accession PXD014791, and they are freely available for the research community^20^. Online-only Table 2 provides the metadata information of drug treatments, cell lines and replication numbers for all raw mass spectrometry data files. In addition, the processed higher level data is available through LINCS Data Portal (http://lincsportal.ccs.miami.edu/dcic-portal/) with the accession LDG-1444. Data include metadata, raw files of LC-MS/MS analysis, protein FASTA database, and protein identification and quantitation results from MaxQuant^21^.

## Technical Validation

We have developed a three-tiered quality control strategy to ensure the high throughput production of deep and reproducible DToxS proteomics datasets. This strategy, which focuses on samples, instrument and analytical pipeline, has enabled us to ensure high quality at (1) the protein sample level, (2) the LC-MS/MS instrument performance level, and (3) the overall quantitative data processing variations.

### Quality validation of the protein samples for LC-MS/MS

Due to both the cost constraints of treating the human primary cardiomyocyte-like cells with KI drugs and the scientific necessity to compare proteomics data with RNAseq data from the same samples, the same drug-treated cells were used to analyze protein expression, after the RNA extraction. The challenges from this approach included occasionally uneven or poor protein yields, and imprecise protein concentration estimates with the established Bradford or BCA assays, due to the confounding components in the RNA extraction buffers. While the protein yield of our combined RNA-protein extraction approach was lower and more variable compared to standard “protein-only” protocols, we note that all of the samples investigated here had more than adequate protein yield necessary for LC-MS/MS. In order to rigorously compare drug effects with the given sample-to-sample variability in experimental processing, we performed an additional SDS-PAGE in-gel quantification step with a consistent positive control sample and internally-prepared HeLa protein lysates. These gels further served as an additional quality control step, as they were checked for signs of protein degradation (such as smearing), which were not observed in any of the samples. Seventy SDS-PAGE gels were run to increase the estimation accuracy of the proteins derived from the cells (see examples in Supplementary Fig. 1); in each gel, 25 µg of HeLa cell protein lysate was included in the first lane, as the reference for both accurate protein estimation and efficient in-gel digestion, and later for LC-MS/MS data normalization. In brief, the densities of the CBB for each KI-treated cell line in each gel lane were recorded (see examples in Online-only Table 1). The protein amounts from each sample were extrapolated by comparing its CBB density to that of the 25 µg of the internally-prepared HeLa proteins; the estimated protein amounts were later used to obtain 0.5 µg/µL equivalent of the tryptic peptides from each sample for LC-MS/MS analysis (see examples in Fig. 2a). For tryptic digestion, the drug-treated samples were digested along with the internally-prepared HeLa proteins separated on the same SDS-PAGE gels, which were later used for LC-MS/MS data normalization as well.

### Quality validation of the LC-MS/MS system

**Fig. 3:**
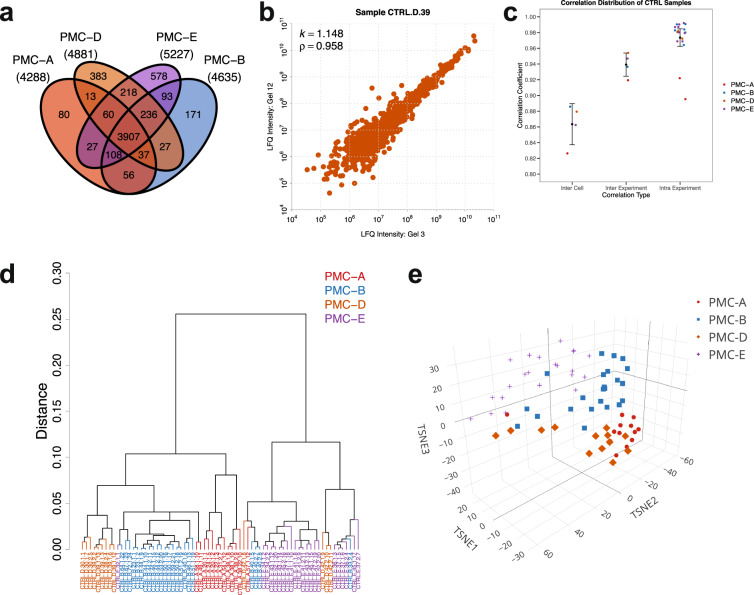
Coverage and reproducibility of detected proteins in control samples. (**a**) Venn diagram of the overlapping and unique protein groups across all samples for the four cardiomyocyte lines PMC-A, B, D, E. (**b**) Normalized MS1 LFQ intensities for the same sample measured from two separate SDS-PAGE gels in two different MS/MS runs had strong agreement. (**c**) The correlation (mean and variation) of LFQ intensity between the control samples from the same experiment for each experiment versus the samples from different experiments in each cell line (cell lines PMC-A, B, D, E shown in color coordination). (**d**) The clustering of all biological replicates from four cell lines under control conditions based on the Eucledian distances of LFQ intensities of replicates. (**e**) Accordingly, proteomic signatures of the four cell lines under control conditions across 71 biological replicates showed strong clustering based on the source cell line.

To ensure that LC-MS/MS system could deliver overall satisfactory performance, 200 ng of commercial HeLa cell digest (Pierce, Cat# 88329) was run prior to each gel batch of the biological samples. To confirm well-behaved LC separations, MS1 ions for select peptides were carefully monitored for retention time shifts and peak widths. In order to minimize the run-to-run carry-over of peptides, a duo-LC system was configured in parallel for this study, so while one LC column was used for peptides separation and LC-MS/MS analysis, the other LC column could be washed to remove any residual peptides. For the overall LC-MS/MS quality control, the system is capable of identifying >3,800 unique proteins and >14,000 unique peptides from ~200 ng of the commercial HeLa cell digest. Indeed, the Levey-Jennings quality assurance plot showing the monthly total ion current (TIC) of the HeLa digest indicates consistent LC-MS/MS system performance for LFQ quantification (Fig. 2b). Qualitatively, the LC-MS/MS system enabled the deep proteome coverages (~5,000 unique protein groups/cell line) of all four DToxS cell lines (Fig. 3a and Table 3). Total 372 raw LC-MS/MS files, including the files for 308 drug treated cardiomyocyte-like cell line and 64 HeLa cell line samples, were submitted to the MaxQuant analysis (Table 3). In total, 83,674 unique peptides that mapped onto 5,994 unique protein groups were identified with the FDR of less than 1%, among the four different cell lines. These proteins were mapped onto 5,784 unique genes. Given no multi-dimensional LC fractionation was performed, these deep proteome coverages confirm the effectiveness of the LC-MS/MS quality control procedures used for this study.

### Quality control of the quantitative data analysis

To minimize the LC-MS/MS quantification noise derived from the sample preparation steps, the HeLa proteins used for the in-gel protein estimation were digested along with the drug-treated samples from the same gels. Thus, these HeLa cell protein digests were used to control for the trypsin digestion efficiencies, LC-MS/MS variations and for the quantitative normalization of the LFQ data. For example, the TIC analyses indicates sample-to-sample variations among both HeLa and control (i.e., no drug treatment) cell digests (Fig. 2c,upper and lower panels, respectively); with HeLa TIC signal normalization, the variations in the control cell digests were further reduced (Fig. 2d). We also randomized all samples into separate gels and included multiple technical replicates of control samples into the gels to prevent time-dependent artifacts that may arise from instrumental drift or lot-to-lot variations in reagents.

Overall, our method for protein count normalization showed good reproducibility (Fig. 3b), which can be quantitatively assessed using Pearson correlation across two technical replicates that were analyzed on different days from two different HeLa signal normalization protocols (i.e., different SDS-PAGE gels). This was consistent for all technical replicates in this dataset (Supplementary Fig. 2). In fact, the average slope for sample-to-sample comparison was remarkably close to unity: 1.03 ± 0.16. Similarly, the average coefficient of determination for all technical replicates in the study was 0.97 ± 0.03. We also note that both the depth of protein quantification for each replicate and the overlap of detected proteins between different biological replicates were consistently high for all cardiomyocyte-like cell lines (Fig. 3c), suggesting that our experimental pipeline was robust in identification of proteomic signatures. The quantification strategy was also successful in reproducible identification of subtle proteomic differences between individual cardiomyocyte cell lines PMC-A, B, D and E, which clustered consistently across most biological replicates using either Pearson (Fig. 3d) or Euclidean (Fig. 3e) distance metrics. Furthermore, we used overlap analysis, to show that most of the identified protein groups were common to either all four cell lines (3,907 or 65%) or detected in at least three out of four lines (4,348 or 73%) with only 1,212 (or 20%) protein groups detected in uniquely one of the lines (Fig. 3a).

### Identification of differentially expressed proteins

The normalized LFQ intensities of identified proteins were processed by a custom computational pipeline for identifying differentially expressed proteins (DEPs) that consists of four parts: (1) general logarithmic transformation and variance stabilization; (2) linear model fitting for treatment comparison; (3) empirical Bayes test for differential statistical significance; and (4) Fisher’s test for combining experiment-wise differential comparison results (Supplementary Fig. 3).

First, for each experiment on each cell line for each drug treatment, the LFQ intensity measurements of drug-treated and control conditions are transformed with a general logarithm formula based on inverse hyperbolic sine function using the *vsnMatrix* and *predict* function of the *vsn* R package such that the variance of transformed data becomes independent of its mean value. Then the transformed intensities of the samples are fitted to the linear model and the fitting coefficients are estimated using the *lmFit* and *contrasts*.fit functions from the *limma* R package. Next, these fitting parameters are given to the empirical Bayes test using the *eBayes* function with its intensity-based trend parameter turned on (for the purpose of dynamic prior estimation) to calculate various statistical metrics for the differential comparison between the drug-treated samples and the control samples. Finally, the p-values from all individual independent differential comparisons are combined using the Fisher’s method with the *sumlog* function of the *metap* R package to obtain a single meta p-value for the comparison between all the drug-treated samples and the control samples for a cell line and a drug treatment. This procedure is then repeated for all the combinations of cell lines and drug treatments. This produces a list of reference proteins ranked by their p-values (from most to least significant). These ranked protein lists together with their cell lines and treated drugs are passed to the downstream analysis.

### Downstream analysis of differentially expressed proteins

**Fig. 4:**
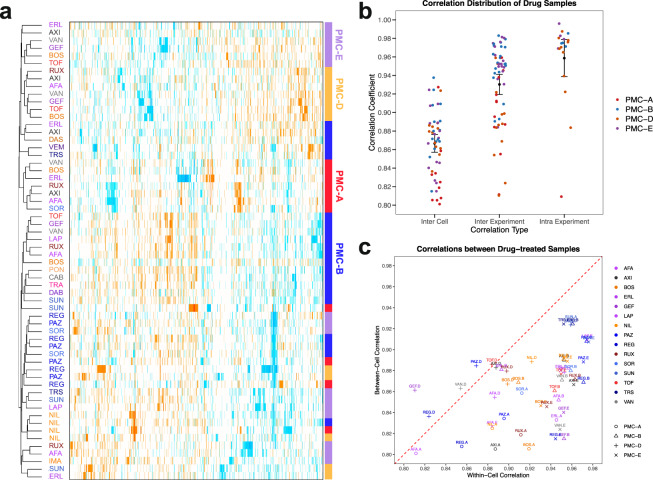
Reproducibility of differential protein expression measurements for drug-treated samples. (**a**) Unsupervised clustering of top 100 differentially expressed proteins in KI treated cardiomyocyte-like cell lines. Color-coding on the left reflects the primary target of the drugs (see Table 1 for further details), whereas the bars on the right highlight the cell line. While KIs with weak proteomic signatures cluster along cell lines, those that have strong differential protein expression cluster along the primary target profile of the drugs. (**b**) The similarity and variability of drug-treated samples within and between cell lines as determined by Pearson correlation between drug treated replicates and controls. (**c**) A summary of the drugs showing similar profiles between cells versus those showing different profiles within cells.

To better collate the cell line and drug specific responses, we first merged all DEPs of the same cell line treated with the same drug to one new list of DEPs. For each DEP list, we then calculated a combined p-value defined as the geometric mean of the individual p-values of the different treatments and a combined log_2_(fold changes) defined as the average mean of the individual log_2_(fold changes). For all downstream analyses, we considered the top 100 DEPs (based on the combined p-values). Combined drug treatments of the different cell lines were hierarchically clustered based on the Euclidean distance of the combined log_2_(fold changes) using Cluster 3.0^22,23^ (Fig. 4a). In order to investigate the consistency between the different experiments for the same drug treatment across the four cell lines, we ran successive Pearson correlations between different replicates for a given drug treatment of the same cell compared to the correlation among different cell types (Fig. 4b).

## Usage Notes

### Use case 1: Identification of KI drug-induced protein expression profiles

This DToxS dataset can be mined to identify protein expression changes triggered by each KI drug treatment of the four cardiac cell lines derived from human tissues. For example, we note that the DEP signatures from different cell lines and KIs clustered together based on the impact of the drug. While KIs that had modest proteomic changes clustered along the cell lines (top clusters of Fig. 4a), those that have more substantial differential protein expression signatures (bottom clusters in Fig. 4a that include nilotinib, sorafenib, pazopanib and regorafenib, all of which are reported to have cardiotoxic adverse associations including QT prolongation and myocardial infarction) clustered on their own irrespective of the cell line. The proteomic changes identified in this study can be used to understand the cell signal transduction events that may underlie KI-induced cardiotoxicities. Also, with the LC-MS/MS information provided in this dataset, follow-up proteomics assays can be developed to validate the protein expression changes in larger number of samples; such assays may include either targeted assays, *e.g*., selected or parallel reaction monitoring or untargeted proteomics assays, *e.g*., data-independent analysis.

### Use case 2: Variability of differential KI drug-induced protein expression profiles

This dataset can be analyzed to compare and cluster the proteomic changes induced by different KIs, thus ascertaining the similarities and idiosyncrasies of different drugs and patient (or cell line) specific responses. One can also quantify the variability and consistency of drug-induced proteomic changes within a given subject (i.e., within a given cell line) compared across a population (i.e., between lines) to determine the generalizability of a given drug effect on cardiomyocytes (Fig. 4c).

### Use case 3: Integration of transcript and protein expression profiles for comprehensive network studies

Drugs may induce complex changes on either gene or protein expression. Since proteomic and transcriptomic changes in complex human cells are not always in agreement (due to technical bias, differential regulation or turnover rates), it may be desirable to integrate both gene and protein expression datasets from the same samples, in order to obtain more comprehensive understanding of the drug-induced changes among the signaling networks. For this purpose, the DToxS proteomics dataset presented here can be integrated with the DToxS transcriptomics dataset^17^ to fully understand the effects of KI-induced cardiotoxicities. We note that 97% of the protein groups in our dataset map to individual genes (Supplementary Fig. 4).

### Use case 4: Identification of drug-induced post-translational modifications (PTMs) and regulated proteolysis

Drugs may induce changes among signaling pathways not only *via* the changes of gene and protein expressions, but also *via* the rapid regulations of protein functions by altering specific PTMs (*e.g*., phosphorylation) and regulated proteolytic events (*e.g*., caspase 3 activation)^24–26^. Therefore, alternative protein database search schemes can be carried out to analyze the raw DToxS proteomics dataset (Supplementary Fig. 5a), and identify KI-induced changes among specific PTMs (Supplementary Fig. 5b,c) or semi-tryptic or non-tryptic peptides derived from regulated proteolysis. Since the current workflow did not include the enrichments of the subproteomes specific for PTMs, the yield for peptides containing PTMs or non-tryptic cleavages will likely be modest. As such, the results from the alternative analysis of the DToxS dataset may provide information for future proteomics studies that focus on the enrichments of specific PTMs, *e.g*. acetylation and phosphorylation.

## Supplementary information

Supplementary Information

## Data Availability

All of the annotated custom code and scripts used for the generation of this dataset is publicly available at the DToxS GitHub repository at https://github.com/DToxS.

## Online-only Tables

**Online-only Table 1:** Metrics of protein quantification via serial SDS-PAGE.

Gel 1	Density	Ratio	Protein Amount (μg)	Resuspend Volume(ul)	Conc.(ug/ul)
HeLa	4851	1	25	50	0.5
CTRL	3121	0.64	16	32.2	0.5
ERL	2289	0.47	12	23.6	0.5
BOS	2099	0.43	11	21.6	0.5
VSN	2003	0.41	10	20.6	0.5
NIL	3498	0.72	18	36.1	0.5
**Gel 2**	**Density**	**Ratio**	**Protein Amount** (**μg**)	**Resuspend Volume**(**ul**)	**Conc**.(**ug/ul**)
HeLa	8468	1	25	50	0.5
CTRL	3622	0.43	11	21.4	0.5
AXI	5688	0.67	17	33.6	0.5
PAZ	5929	0.7	18	35	0.5
RUX	6428	0.76	19	38	0.5
AFA	7152	0.84	21	42.2	0.5
**Gel 3**	**Density**	**Ratio**	**Protein Amount** (**μg**)	**Resuspend Volume**(**ul**)	**Conc**.(**ug/ul**)
HeLa	8468	1	25	50	0.5
CTRL	3622	0.43	11	21.4	0.5
AXI	5688	0.67	17	33.6	0.5
PAZ	5929	0.7	18	35	0.5
RUX	6428	0.76	19	38	0.5
AFA	7152	0.84	21	42.2	0.5
**Gel 4**	**Density**	**Ratio**	**Protein Amount** (**μg**)	**Resuspend Volume**(**ul**)	**Conc**.(**ug/ul**)
HeLa	9789	1	25	50	0.5
NIL	5914	0.6	15	30.2	0.5
AXI	7570	0.77	19	38.7	0.5
PAZ	7507	0.77	19	38.3	0.5
RUX	7172	0.73	18	36.6	0.5
AFA	6802	0.69	17	34.7	0.5
**Gel 5**	**Density**	**Ratio**	**Protein Amount** (**μg**)	**Resuspend Volume**(**ul**)	**Conc**.(**ug/ul**)
HeLa	8720	1	25	50	0.5
ERL	5983	0.69	17	34.3	0.5
SOR	6080	0.7	18	34.9	0.5
BOS	6559	0.75	19	37.6	0.5
VSN	6436	0.74	19	36.9	0.5
**Gel 6**	**Density**	**Ratio**	**Protein Amount** (**μg**)	**Resuspend Volume**(**ul**)	**Conc**.(**ug/ul**)
HeLa	2337	1	25	50	0.5
NIL	1988	0.85	21	42.5	0.5
AXI	1660	0.71	18	35.5	0.5
PAZ	1844	0.79	20	39.5	0.5
RUX	1757	0.75	19	37.6	0.5
AFA	1895	0.81	20	40.5	0.5
**Gel 7**	**Density**	**Ratio**	**Protein Amount** (**μg**)	**Resuspend Volume**(**ul**)	**Conc**.(**ug/ul**)
HeLa	3409	1	25	50	0.5
CTRL	2767	0.81	20	40.6	0.5
SOR	2006	0.59	15	29.4	0.5
**Gel 8**	**Density**	**Ratio**	**Protein Amount** (**μg**)	**Resuspend Volume**(**ul**)	**Conc**.(**ug/ul**)
HeLa	2439	1	25	50	0.5
CTRL	1786	0.73	18	36.6	0.5
BOS	1878	0.77	19	38.5	0.5
VAN	2200	0.9	23	45.1	0.5
NIL	1953	0.8	20	40	0.5
AXI	2096	0.86	22	43	0.5
**Gel 9**	**Density**	**Ratio**	**Protein Amount** (**μg**)	**Resuspend Volume**(**ul**)	**Conc**.(**ug/ul**)
HeLa	2708	1	25	50	0.5
CTRL	2282	0.84	21	42.1	0.5
RUX	2144	0.79	20	39.6	0.5
AFA	2199	0.81	20	40.6	0.5
REG	2965	1.09	27	54.7	0.5
REG	2070	0.76	19	38.2	0.5
**Gel 10**	**Density**	**Ratio**	**Protein Amount** (**μg**)	**Resuspend Volume**(**ul**)	**Conc**.(**ug/ul**)
HeLa	2869	1	25	50	0.5
CTRL	2236	0.78	20	39	0.5
SUN	1880	0.66	17	32.8	0.5
CTRL	2262	0.79	20	39.4	0.5
SUN	2240	0.78	20	39	0.5
**Gel 11**	**Density**	**Ratio**	**Protein Amount** (**μg**)	**Resuspend Volume**(**ul**)	**Conc**.(**ug/ul**)
HeLa	2317	1	25	50	0.5
CTRL	1760	0.76	19	38	0.5
PAZ	1321	0.57	14	28.5	0.5
REG	1575	0.68	17	34	0.5
SUN	1888	0.81	20	40.7	0.5
**Gel 12**	**Density**	**Ratio**	**Protein Amount** (**μg**)	**Resuspend Volume**(**ul**)	**Conc**.(**ug/ul**)
HeLa	1444	1	25	50	0.5
CTRL	1121	0.78	20	38.8	0.5
REG	995	0.69	17	34.5	0.5
CTRL	1062	0.74	19	36.8	0.5
SUN	1023	0.71	18	35.4	0.5

**Online-only Table 2:** Drug treatments, cell lines and replication numbers for raw mass spectrometry data files.

Raw Mass Spectrometry File	Drug Treatment	Cell Line	Replicate Number
031616_Gel2_Exp20_AFA_Tube_17.raw	AFA	PMC-A	1
031616_Gel4_AFA_Tube_20_160506081617.raw	AFA	PMC-A	2
031616_Gel6_Exp24_AFA_Tube_23.raw	AFA	PMC-A	3
031616_Gel9_AFA_Exp38_Tube_10.raw	AFA	PMC-A	4
080616_Gel7_AFA_Exp40_Tube_12.raw	AFA	PMC-B	1
071116_Gel11_AFA_Exp44_Tube_15.raw	AFA	PMC-B	2
093016_Gel_14_AFA_Exp46_Tube14_161004062123.raw	AFA	PMC-B	3
091216_Gel17_AFA_Exp50_Tube_10.raw	AFA	PMC-B	4
Gel_5_Experiment_30_AFA_150923052026.raw	AFA	PMC-D	1
Gel_6_Experiment_39_AFA_150926090401.raw	AFA	PMC-D	2
Gel_12_Experiment_39_AFA1.raw	AFA	PMC-D	3
Gel_9_Experiment_42_AFA1.raw	AFA	PMC-D	4
Evren_Gel7_Tube11_AFA_Exp_37_2000ng.raw	AFA	PMC-E	1
Evren_Gel10_Tube14_AFA_Exp_41_3000ng.raw	AFA	PMC-E	2
Evren_Gel10_Tube15_AFA_Exp_41_3000ng.raw	AFA	PMC-E	3
Gel13_Tube10_AFA_Exp_43.raw	AFA	PMC-E	4
Gel_19_Tube18_AFA_Exp_47.raw	AFA	PMC-E	5
031616_Gel2_Exp20_AXI_Tube_14_160504082926.raw	AXI	PMC-A	1
031616_Gel4_AXI_Tube_17_160505201521.raw	AXI	PMC-A	2
031616_Gel6_Exp24_AXI_Tube_20.raw	AXI	PMC-A	3
031616_Gel8_AXI_Exp38_Tube7.raw	AXI	PMC-A	4
080216_Gel6_AXI_Exp40_Tube_9.raw	AXI	PMC-B	1
071116_Gel10_AXI_Exp44_Tube_12.raw	AXI	PMC-B	2
093016_Gel_14_AXI_Exp46_Tube11.raw	AXI	PMC-B	3
091216_Gel16_AXI_Exp50_Tube_7.raw	AXI	PMC-B	4
Gel_5_Experiment_30_AXI_150922211943.raw	AXI	PMC-D	1
Gel_6_Experiment_39_AXI_150926010323.raw	AXI	PMC-D	2
Gel_12_Experiment_39_AXI1.raw	AXI	PMC-D	3
Gel_9_Experiment_42_AXI1.raw	AXI	PMC-D	4
Evren_Gel4_Tube12_AXI_Exp_36_1500ng.raw	AXI	PMC-E	1
Evren_Gel6_Tube8_AXI_Exp_37_2000ng.raw	AXI	PMC-E	2
Evren_Gel9_Tube10_AXI_Exp_41_3000ng.raw	AXI	PMC-E	3
Gel12_Tube7_AXI_Exp_43.raw	AXI	PMC-E	4
Gel_19_Tube19_AXI_Exp_47.raw	AXI	PMC-E	5
031616_Gel1_BOS_Tube_11.raw	BOS	PMC-A	1
031616_Gel3_Exp22_BOS_Tube_14.raw	BOS	PMC-A	2
031616_Gel5_Exp24_BOS_Tube_17.raw	BOS	PMC-A	3
031616_Gel8_BOS_Exp38_Tube_4.raw	BOS	PMC-A	4
072216_Gel2_BOS_Exp23_Tube_7.raw	BOS	PMC-B	1
072216_Gel2_BOS_Exp23_Tube_8.raw	BOS	PMC-B	2
09082016_Gel4_EXP33_BOS_tube24.raw	BOS	PMC-B	3
071116_Gel10_BOS_Exp44_Tube_9.raw	BOS	PMC-B	4
Gel_7_Experiment_30_BOS1.raw	BOS	PMC-D	1
Gel_13_Experiment_39_BOS_151014135302.raw	BOS	PMC-D	2
Gel_10_Experiment_42_BOS1_151015095436.raw	BOS	PMC-D	3
Gel_11_Experiment_42_BOS1.raw	BOS	PMC-D	4
Evren_Gel3_Tube9_BOS_Exp_36_1500ng.raw	BOS	PMC-E	1
Evren_Gel5_Tube5_BOS_Exp_37_2000ng.raw	BOS	PMC-E	2
Evren_Gel8_Tube7_BOS_Exp_41_2000ng.raw	BOS	PMC-E	3
Gel12_Tube4_BOS_Exp_43.raw	BOS	PMC-E	4
Gel15_Tube10_BOS_Exp_45.raw	BOS	PMC-E	5
080616_Gel8_CAB_Exp40_Tube_19.raw	CAB	PMC-B	1
09082016_Gel12_EXP44_CAB_tube20.raw	CAB	PMC-B	2
091216_Gel15_CAB_Exp46_Tube_19.raw	CAB	PMC-B	3
09232016_Gel18_EXP50_CAB_tube15.raw	CAB	PMC-B	4
080616_Gel8_DAB_Exp40_Tube_17.raw	DAB	PMC-B	1
09082016_Gel12_EXP44_DAB_tube18.raw	DAB	PMC-B	2
091216_Gel15_DAB_Exp46_Tube_17.raw	DAB	PMC-B	3
09192016_Gel19_EXP51_DAB_tube22.raw	DAB	PMC-B	4
080216_Gel5_DAS_Exp40_Tube_5.raw	DAS	PMC-B	1
071116_Gel9_DAS_Exp44_Tube_3.raw	DAS	PMC-B	2
093016_Gel_13_DAS_Exp46_Tube3.raw	DAS	PMC-B	3
091216_Gel16_DAS_Exp50_Tube_3.raw	DAS	PMC-B	4
031616_Gel1_ERL_Tube_5.raw	ERL	PMC-A	1
031616_Gel5_Exp24_ERL_Tube_8.raw	ERL	PMC-A	2
072716_Gel3_ERL_Exp23_Tube_22.raw	ERL	PMC-B	1
09082016_Gel4_EXP33_ERL_tube10.raw	ERL	PMC-B	2
080216_Gel5_ERL_Exp40_Tube_3.raw	ERL	PMC-B	3
080216_Gel5_ERL_Exp40_Tube_4.raw	ERL	PMC-B	4
Gel_4_Experiment_28_ERL1.raw	ERL	PMC-D	1
Gel_5_Experiment_30_ERL_150922171926.raw	ERL	PMC-D	2
Gel_3_Experiment_39_ERL1.raw	ERL	PMC-D	3
Gel_9_Experiment_42_ERL1.raw	ERL	PMC-D	4
Evren_Gel1_Tube22_ERL_Exp_32_2000ng.raw	ERL	PMC-E	1
Evren_Gel8_Tube3_ERL_Exp_41_2000ng.raw	ERL	PMC-E	2
072216_Gel1_GEF_Exp23_Tube_5.raw	GEF	PMC-B	1
072216_Gel1_GEF_Exp23_Tube_6.raw	GEF	PMC-B	2
080216_Gel5_GEF_Exp40_Tube_6.raw	GEF	PMC-B	3
071116_Gel9_GEF_Exp44_Tube_7.raw	GEF	PMC-B	4
Gel_7_Experiment_30_GEF1.raw	GEF	PMC-D	1
Gel_13_Experiment_39_GEF_151014095241.raw	GEF	PMC-D	2
Gel_10_Experiment_42_GEF1_151015055419.raw	GEF	PMC-D	3
Gel_11_Experiment_42_GEF1.raw	GEF	PMC-D	4
Evren_Gel1_Tube12_GEF_Exp_32_2000ng.raw	GEF	PMC-E	1
Evren_Gel2_Tube11_GEF_Exp_34_2000ng.raw	GEF	PMC-E	2
Evren_Gel3_Tube8_GEF_Exp_36_1500ng.raw	GEF	PMC-E	3
Evren_Gel5_Tube4_GEF_Exp_37_2000ng.raw	GEF	PMC-E	4
Evren_Gel8_Tube6_GEF_Exp_41_2000ng.raw	GEF	PMC-E	5
Evren_Gel1_Tube13_IMA_Exp_32_2000ng.raw	IMA	PMC-E	1
Evren_Gel2_Tube12_IMA_Exp_34_2000ng.raw	IMA	PMC-E	2
Gel15_Tube4_IMA_Exp_45.raw	IMA	PMC-E	3
071116_Gel9_LAP_Exp44_Tube_6.raw	LAP	PMC-B	1
Gel14_Tube18_LAP_Exp_43.raw	LAP	PMC-E	1
Gel_16_Tube12_LAP_Exp_45.raw	LAP	PMC-E	2
Gel_18_Tube6_LAP_Exp_47.raw	LAP	PMC-E	3
031616_Gel1_NIL_Tube_13.raw	NIL	PMC-A	1
031616_Gel4_NIL_Tube_16_160505161502.raw	NIL	PMC-A	2
031616_Gel6_Exp24_NIL_Tube_19.raw	NIL	PMC-A	3
031616_Gel8_NIL_Exp38_Tube_6.raw	NIL	PMC-A	4
080216_Gel6_NIL_Exp40_Tube_8.raw	NIL	PMC-B	1
071116_Gel10_NIL_Exp44_Tube_11.raw	NIL	PMC-B	2
093016_Gel_13_NIL_Exp46_Tube10.raw	NIL	PMC-B	3
091216_Gel16_NIL_Exp50_Tube_6.raw	NIL	PMC-B	4
Gel_1_Experiment_30_NIL_1.raw	NIL	PMC-D	1
2nd_Experiment_39_NIL_1.raw	NIL	PMC-D	2
Gel_3_Experiment_39_NIL1.raw	NIL	PMC-D	3
Gel_8_Experiment_42_NIL1.raw	NIL	PMC-D	4
Evren_Gel4_Tube11_NIL_Exp_36_1500ng.raw	NIL	PMC-E	1
Evren_Gel6_Tube7_NIL_Exp_37_2000ng.raw	NIL	PMC-E	2
Evren_Gel9_Tube9_NIL_Exp_41_3000ng.raw	NIL	PMC-E	3
Gel12_Tube6_NIL_Exp_43.raw	NIL	PMC-E	4
Gel_17_Tube23_NIL_Exp_45.raw	NIL	PMC-E	5
031616_Gel2_Exp20_PAZ_Tube_15.raw	PAZ	PMC-A	1
031616_Gel4_PAZ_Tube_18_160506001539.raw	PAZ	PMC-A	2
031616_Gel6_Exp24_PAZ_Tube_21.raw	PAZ	PMC-A	3
031616_Gel11_PAZ_Exp49_Tube_8_160619012456.raw	PAZ	PMC-A	4
080216_Gel6_PAZ_Exp40_Tube_10.raw	PAZ	PMC-B	1
071116_Gel11_PAZ_Exp44_Tube_13.raw	PAZ	PMC-B	2
093016_Gel_13_PAZ_Exp46_Tube12.raw	PAZ	PMC-B	3
091216_Gel16_PAZ_Exp50_Tube_8.raw	PAZ	PMC-B	4
Gel_1_Experiment_30_PAZ_1.raw	PAZ	PMC-D	1
2nd_Experiment_39_PAZ_1.raw	PAZ	PMC-D	2
Gel_3_Experiment_39_PAZ1.raw	PAZ	PMC-D	3
Gel_8_Experiment_42_PAZ1.raw	PAZ	PMC-D	4
Evren_Gel4_Tube13_PAZ_Exp_36_1500ng.raw	PAZ	PMC-E	1
Evren_Gel6_Tube9_PAZ_Exp_37_2000ng.raw	PAZ	PMC-E	2
Evren_Gel9_Tube11_PAZ_Exp_41_3000ng.raw	PAZ	PMC-E	3
Gel13_Tube8_PAZ_Exp_43.raw	PAZ	PMC-E	4
Gel_17_Tube21_PAZ_Exp_45.raw	PAZ	PMC-E	5
080616_Gel7_PON_Exp40_Tube_14.raw	PON	PMC-B	1
09082016_Gel12_EXP44_PON_tube17.raw	PON	PMC-B	2
091216_Gel15_PON_Exp46_Tube_16.raw	PON	PMC-B	3
091216_Gel17_PON_Exp50_Tube_12.raw	PON	PMC-B	4
031616_Gel9_REG_Exp38_Tube_11.raw	REG	PMC-A	1
031616_Gel9_REG_Exp38_Tube_12.raw	REG	PMC-A	2
031616_Gel11_REG_Exp49_Tube_9_160619052515.raw	REG	PMC-A	3
031616_Gel12_REG_Exp54_Tube_9_160619212635.raw	REG	PMC-A	4
080616_Gel7_REG_Exp40_Tube_13.raw	REG	PMC-B	1
071116_Gel11_REG_Exp44_Tube_16.raw	REG	PMC-B	2
093016_Gel_14_REG_Exp46_Tube15.raw	REG	PMC-B	3
091216_Gel17_REG_Exp50_Tube_11.raw	REG	PMC-B	4
Gel_1_Experiment_30_REG_1.raw	REG	PMC-D	1
2nd_Experiment_39_REG_1.raw	REG	PMC-D	2
Gel_3_Experiment_39_REG1.raw	REG	PMC-D	3
Gel_8_Experiment_42_REG1.raw	REG	PMC-D	4
Evren_Gel7_Tube12_REG_Exp_37_2000ng.raw	REG	PMC-E	1
Evren_Gel11_Tube16_REG_Exp_41_3000ng.raw	REG	PMC-E	2
Evren_Gel11_Tube17_REG_Exp_41_3000ng.raw	REG	PMC-E	3
Gel13_Tube11_REG_Exp_43.raw	REG	PMC-E	4
Gel_17_Tube20_REG_Exp_45.raw	REG	PMC-E	5
031616_Gel2_Exp20_RUX_Tube_16.raw	RUX	PMC-A	1
031616_Gel4_RUX_Tube_19_160506041556.raw	RUX	PMC-A	2
031616_Gel6_Exp24_RUX_Tube_22.raw	RUX	PMC-A	3
031616_Gel9_RUX_Exp38_Tube_9.raw	RUX	PMC-A	4
080616_Gel7_RUX_Exp40_Tube_11.raw	RUX	PMC-B	1
071116_Gel11_RUX_Exp44_Tube_14.raw	RUX	PMC-B	2
093016_Gel_14_RUX_Exp46_Tube13.raw	RUX	PMC-B	3
091216_Gel17_RUX_Exp50_Tube_9.raw	RUX	PMC-B	4
Gel_5_Experiment_30_RUX_150923012006.raw	RUX	PMC-D	1
Gel_6_Experiment_39_RUX_150926050342.raw	RUX	PMC-D	2
Gel_12_Experiment_39_RUX1.raw	RUX	PMC-D	3
Gel_9_Experiment_42_RUX1.raw	RUX	PMC-D	4
Evren_Gel6_Tube10_RUX_Exp_37_2000ng.raw	RUX	PMC-E	1
Evren_Gel10_Tube12_RUX_Exp_41_3000ng.raw	RUX	PMC-E	2
Evren_Gel10_Tube13_RUX_Exp_41_3000ng.raw	RUX	PMC-E	3
Gel13_Tube9_RUX_Exp_43.raw	RUX	PMC-E	4
Gel_19_Tube17_RUX_Exp_47.raw	RUX	PMC-E	5
031616_Gel3_Exp22_SOR_Tube_9.raw	SOR	PMC-A	1
031616_Gel5_Exp24_SOR_Tube_11.raw	SOR	PMC-A	2
031616_Gel7_SOR_Exp31_Tube_16_160620172811.raw	SOR	PMC-A	3
071116_Gel20_SOR_Exp53_tube_20.raw	SOR	PMC-B	1
09022016_Gel23_EXP67_SOR_tube15.raw	SOR	PMC-B	2
09232016_Gel24_EXP72_SOR_tube15.raw	SOR	PMC-B	3
Gel_16_Tube18_SOR_Exp_45.raw	SOR	PMC-E	1
Gel_18_Tube10_SOR_Exp_47.raw	SOR	PMC-E	2
Gel_20_Tube8_SOR_Exp_52.raw	SOR	PMC-E	3
Gel_21_Tube20_SOR_Exp_55.raw	SOR	PMC-E	4
031616_Gel11_SUN_Exp49_Tube_19_160619092535.raw	SUN	PMC-A	1
031616_Gel12_SUN_Exp57_Tube_4_160620052717.raw	SUN	PMC-A	2
031616_Gel10_SUN_Exp58_Tube_6.raw	SUN	PMC-A	3
09232016_Gel18_EXP50_SUN_tube18.raw	SUN	PMC-B	1
09192016_Gel19_EXP51_SUN_tube9.raw	SUN	PMC-B	2
071116_Gel20_SUN_Exp53_tube_4.raw	SUN	PMC-B	3
09022016_Gel21_EXP59_SUN_tube3.raw	SUN	PMC-B	4
Gel_4_Experiment_28_SUN1.raw	SUN	PMC-D	1
Gel_1_Experiment_30_SUN_1.raw	SUN	PMC-D	2
2nd_Experiment_39_SUN_1.raw	SUN	PMC-D	3
Gel_8_Experiment_42_SUN1.raw	SUN	PMC-D	4
Gel14_Tube23_SUN_Exp_43.raw	SUN	PMC-E	1
Gel15_Tube7_SUN_Exp_45.raw	SUN	PMC-E	2
Gel15_Tube8_SUN_Exp_45.raw	SUN	PMC-E	3
Gel_18_Tube4_SUN_Exp_47.raw	SUN	PMC-E	4
Gel_20_Tube4_SUN_Exp_52.raw	SUN	PMC-E	5
Gel_21_Tube10_Sun_Exp_55.raw	SUN	PMC-E	6
072216_Gel1_TOF_Exp23_Tube_3.raw	TOF	PMC-B	1
072216_Gel1_TOF_Exp23_Tube_4.raw	TOF	PMC-B	2
071116_Gel9_TOF_Exp44_Tube_8.raw	TOF	PMC-B	3
093016_Gel_13_TOF_Exp46_Tube9.raw	TOF	PMC-B	4
Gel_7_Experiment_30_TOF1.raw	TOF	PMC-D	1
Gel_13_Experiment_39_TOF1_151014055215.raw	TOF	PMC-D	2
Gel_10_Experiment_42_TOF1_151015015403.raw	TOF	PMC-D	3
Gel_11_Experiment_42_TOF1.raw	TOF	PMC-D	4
Evren_Gel1_Tube11_TOF_Exp_32_2000ng.raw	TOF	PMC-E	1
Evren_Gel3_Tube7_TOF_Exp_36_1500ng.raw	TOF	PMC-E	2
Evren_Gel5_Tube3_TOF_Exp_37_2000ng.raw	TOF	PMC-E	3
Evren_Gel8_Tube5_TOF_Exp_41_2000ng.raw	TOF	PMC-E	4
080616_Gel8_TRA_Exp40_Tube_20.raw	TRA	PMC-B	1
09082016_Gel12_EXP44_TRA_tube21.raw	TRA	PMC-B	2
091216_Gel15_TRA_Exp46_Tube_20.raw	TRA	PMC-B	3
09232016_Gel18_EXP50_TRA_tube16.raw	TRA	PMC-B	4
09022016_Gel21_EXP59_TRS_tube11.raw	TRS	PMC-B	1
09022016_Gel22_EXP64_TRS_tube8.raw	TRS	PMC-B	2
09022016_Gel23_EXP67_TRS_tube8.raw	TRS	PMC-B	3
09232016_Gel24_EXP72_TRS_tube8.raw	TRS	PMC-B	4
Gel14_Tube21_TRS_Exp_43.raw	TRS	PMC-E	1
Gel_16_Tube15_TRS_Exp_45.raw	TRS	PMC-E	2
Gel_18_Tube8_TRS_Exp_47.raw	TRS	PMC-E	3
Gel_21_Tube3_TRS_Exp_55.raw	TRS	PMC-E	4
031616_Gel1_USN_Tube_12.raw	VAN	PMC-A	1
031616_Gel3_Exp22_VAN_Tube_15.raw	VAN	PMC-A	2
031616_Gel5_Exp24_VAN_Tube_18.raw	VAN	PMC-A	3
031616_Gel8_VAN_Exp38_Tube_5.raw	VAN	PMC-A	4
072716_Gel3_VAN_Exp23_Tube_21.raw	VAN	PMC-B	1
080216_Gel6_VAN_Exp40_Tube_7.raw	VAN	PMC-B	2
071116_Gel10_VAN_Exp44_Tube_10.raw	VAN	PMC-B	3
Gel_7_Experiment_30_VAN1.raw	VAN	PMC-D	1
Gel_13_Experiment_39_VAN_151014175319.raw	VAN	PMC-D	2
Gel_10_Experiment_42_VAN1_151015135454.raw	VAN	PMC-D	3
Gel_11_Experiment_42_VAN1.raw	VAN	PMC-D	4
Evren_Gel3_Tube10_VAN_Exp_36_1500ng.raw	VAN	PMC-E	1
Evren_Gel5_Tube6_VAN_Exp_37_2000ng.raw	VAN	PMC-E	2
Evren_Gel9_Tube8_VAN_Exp_41_3000ng.raw	VAN	PMC-E	3
Gel12_Tube5_VAN_Exp_43.raw	VAN	PMC-E	4
Gel_16_Tube11_VAN_Exp_45.raw	VAN	PMC-E	5
09022016_Gel21_EXP59_VEM_tube15.raw	VEM	PMC-B	1
09022016_Gel23_EXP67_VEM_tube2.raw	VEM	PMC-B	2
09232016_Gel24_EXP72_VEM_tube2.raw	VEM	PMC-B	3
031616_Gel1_Ctrl_Tube_1.raw	CTRL	PMC-A	1
031616_Gel2_Exp20_Ctrl_Tube_1_160430071114.raw	CTRL	PMC-A	2
031616_Gel3_Exp22_CTRL_Tube_1.raw	CTRL	PMC-A	3
031616_Gel5_Exp24_CTRL_Tube_1.raw	CTRL	PMC-A	4
031616_Gel7_CTRL_Exp31_Tube_2_160620132755.raw	CTRL	PMC-A	5
031616_Gel8_CTRL_Exp38_Tube_1.raw	CTRL	PMC-A	6
031616_Gel10_CTRL_Exp38_Tube_1.raw	CTRL	PMC-A	7
031616_Gel9_CTRL_Exp38_Tube_2_160518133038.raw	CTRL	PMC-A	8
031616_Gel10_SUN_Exp38_Tube_2.raw	CTRL	PMC-A	9
031616_Gel11_CTRL_Exp49_Tube_1_160618212423.raw	CTRL	PMC-A	10
031616_Gel12_CTRL_Exp54_Tube_1_160619172619.raw	CTRL	PMC-A	11
031616_Gel12_CTRL_Exp57_Tube_1_160620012658.raw	CTRL	PMC-A	12
031616_Gel10_CTRL_Exp58_Tube_1.raw	CTRL	PMC-A	13
072216_Gel1_CTRL_Exp23_Tube_1.raw	CTRL	PMC-B	1
072216_Gel2_CTRL_Exp23_Tube_1.raw	CTRL	PMC-B	2
09082016_Gel4_EXP33_CTRL_tube2.raw	CTRL	PMC-B	3
080216_Gel5_CTRL_Exp40_Tube_1.raw	CTRL	PMC-B	4
080216_Gel6_CTRL_Exp40_Tube_1_160805053123.raw	CTRL	PMC-B	5
080616_Gel7_CTRL_Exp40_Tube_2.raw	CTRL	PMC-B	6
080616_Gel8_CTRL_Exp40_Tube_2.raw	CTRL	PMC-B	7
071116_Gel9_CTRL_Exp44_tube_1.raw	CTRL	PMC-B	8
071116_Gel10_CTRL_Exp44_Tube_1.raw	CTRL	PMC-B	9
071116_Gel11_CTRL_Exp44_Tube_2.raw	CTRL	PMC-B	10
09082016_Gel12_EXP44_CTRL_tube2_160916180753.raw	CTRL	PMC-B	11
093016_Gel_13_CTRL_Exp46_Tube1.raw	CTRL	PMC-B	12
091216_Gel15_CTRL_Exp46_Tube_1.raw	CTRL	PMC-B	13
091216_Gel16_CTRL_Exp50_Tube_1.raw	CTRL	PMC-B	14
091216_Gel17_CTRL_Exp50_Tube_1.raw	CTRL	PMC-B	15
09232016_Gel18_EXP50_CTRl_tube2.raw	CTRL	PMC-B	16
09192016_Gel19_EXP51_CTRL_tube1.raw	CTRL	PMC-B	17
09192016_Gel19_EXP51_CTRL_tube2.raw	CTRL	PMC-B	18
071116_Gel20_CTRL_Exp53_tube_2.raw	CTRL	PMC-B	19
09022016_Gel21_EXP59_CTRL_tube2.raw	CTRL	PMC-B	20
09022016_Gel22_EXP64_CTRL_tube1.raw	CTRL	PMC-B	21
09022016_Gel23_EXP67_CTRL_tube1.raw	CTRL	PMC-B	22
09232016_Gel24_EXP72_CTRL_tube1.raw	CTRL	PMC-B	23
Gel_4_Experiment_28_CTRL1.raw	CTRL	PMC-D	1
Gel_1_Experiment_30_CTRL_1.raw	CTRL	PMC-D	2
Gel_7_Experiment_30_CTRL1.raw	CTRL	PMC-D	3
Gel_5_Experiment_30_CTRL1_150922131908.raw	CTRL	PMC-D	4
Gel_3_Experiment_39_CTRL1.raw	CTRL	PMC-D	5
Gel_6_Experiment_39_CTRL1_150925170228.raw	CTRL	PMC-D	6
Gel_12_Experiment_39_CTRL1.raw	CTRL	PMC-D	7
Gel_13_Experiment_39_CTRL1_151014015157.raw	CTRL	PMC-D	8
2nd_Experiment_39_CTRL_1.raw	CTRL	PMC-D	9
Gel_8_Experiment_42_CTRL1.raw	CTRL	PMC-D	10
Gel_9_Experiment_42_CTRL1.raw	CTRL	PMC-D	11
Gel_10_Experiment_42_CTRL1_151014215338.raw	CTRL	PMC-D	12
Gel_11_Experiment_42_CTRL1.raw	CTRL	PMC-D	13
Evren_Gel1_Tube1_CTRL_Exp_32_2000ng.raw	CTRL	PMC-E	1
Evren_Gel2_Tube1_CTRL_Exp_34_2000ng.raw	CTRL	PMC-E	2
Evren_Gel2_Tube2_CTRL_Exp_34_2000ng.raw	CTRL	PMC-E	3
Evren_Gel3_Tube1_CTRL_Exp_36_1500ng.raw	CTRL	PMC-E	4
Evren_Gel4_Tube2_CTRL_Exp_36_1500ng.raw	CTRL	PMC-E	5
Evren_Gel5_Tube1_CTRL_Exp_37_2000ng.raw	CTRL	PMC-E	6
Evren_Gel6_Tube1_CTRL_Exp_37_2000ng.raw	CTRL	PMC-E	7
Evren_Gel7_Tube2_CTRL_Exp_37_2000ng.raw	CTRL	PMC-E	8
Evren_Gel8_Tube1_CTRL_Exp_41_2000ng.raw	CTRL	PMC-E	9
Evren_Gel10_Tube1_CTRL_Exp_41_3000ng.raw	CTRL	PMC-E	10
Evren_Gel9_Tube2_CTRL_Exp_41_3000ng.raw	CTRL	PMC-E	11
Evren_Gel11_Tube2_CTRL_Exp_41_3000ng.raw	CTRL	PMC-E	12
Gel12_Tube1_CTRL_Exp_43.raw	CTRL	PMC-E	13
Gel14_Tube1_Ctrl_Exp_43.raw	CTRL	PMC-E	14
Gel13_Tube2_CTRL_Exp_43.raw	CTRL	PMC-E	15
Gel15_Tube1_Ctrl_Exp_45.raw	CTRL	PMC-E	16
Gel_17_Tube1_CTRL_Exp_45.raw	CTRL	PMC-E	17
Gel_16_Tube2_CTRL_Exp_45.raw	CTRL	PMC-E	18
Gel_18_Tube1_CTRL_Exp_47.raw	CTRL	PMC-E	19
Gel_19_Tube2_CTRL_Exp_47.raw	CTRL	PMC-E	20
Gel_20_Tube1_CTRL_Exp_52.raw	CTRL	PMC-E	21
Gel_20_Tube2_CTRL_Exp_52.raw	CTRL	PMC-E	22
