# Supplementary material for: Proteomic cellular signatures of kinase inhibitor-induced cardiotoxicity
Source: Sci Data. 2022 Jan 20;9:18. doi: 10.1038/s41597-021-01114-3 (PMC8776854; doi:10.1038/s41597-021-01114-3)
Supplement: Supplementary file 1 — Supplementary Information [file 41597_2021_1114_MOESM1_ESM.pdf]

Supplementary Information

Proteomic cellular signatures of kinase inhibitor-induced cardiotoxicity

*Yuguang Xiong [1, #], Tong Liu [2, #], Tong Chen [2, #], Jens Hansen [1], Bin Hu [1], Yibang Chen [1], Gomathi Jayaraman [1], Stephan Schürer [3, 4], Dusica Vidovic [3, 4], Joseph Goldfarb [1], Eric A. Sobie [1], Marc R. Birtwistle [1, 5], Ravi Iyengar [1, *], Hong Li [2, *] and Evren U. Azeloglu [1, 6, *]*

[1] Department of Pharmacological Sciences and Institute for Systems Biomedicine, Icahn School of Medicine at Mount Sinai, New York, NY 10029
[2] Center for Advanced Proteomics Research and Department of Microbiology, Biochemistry and Molecular Genetics, Rutgers University - New Jersey Medical School, Newark, NJ 07103
[3] Department of Molecular and Cellular Pharmacology, Miller School of Medicine, University of Miami, Miami, FL 33136
[4] Center for Computational Science, University of Miami, Miami, FL 33136
[5] Department of Chemical and Biomolecular Engineering, Clemson University, Clemson, SC 29634
[6] Department of Medicine, Division of Nephrology, Icahn School of Medicine at Mount Sinai, New York, NY 10029

[#] Equal contributions

[*] *Address for correspondence*:
Evren U. Azeloglu, Department of Medicine, Division of Nephrology, Icahn School of Medicine at Mount Sinai, New York, NY 10029. Tel: (212) 241-8519, Fax: (212) 987-0389, Email: evren.azeloglu@mssm.edu
or
Hong Li, Department of Microbiology, Biochemistry and Molecular Genetics, Rutgers University - NJMS, Newark, NJ 07103. Tel: 973-972-8396, Fax: 973-972-1865, E-Mail: liho2@rutgers.edu
or
Ravi Iyengar, Department of Pharmacological Sciences, Icahn School of Medicine at Mount Sinai, New York, NY 10029. Tel: (212) 659-1700, Fax: (212) 831-0114

Supplementary File Table of Contents

Supplementary Figure 1. Examples of protein quantification via serial SDS-PAGE……………………….3

Supplementary Figure 2. Reproducibility of proteomic quantification for technical replicates……………4

Supplementary Figure 3. Workflow for identifying DEPs from LFQ intensities……………………………..6

Supplementary Figure 4. Coverage of identified proteins that map onto genes……………………………7

Supplementary Figure 5. Using the complementary proteomics search methods to identify drug-regulated changes of PTMs……………………………………………………………………………………… 8

Supplementary Table 1: Sample sizes for the KI treated groups per cell line………………………………9

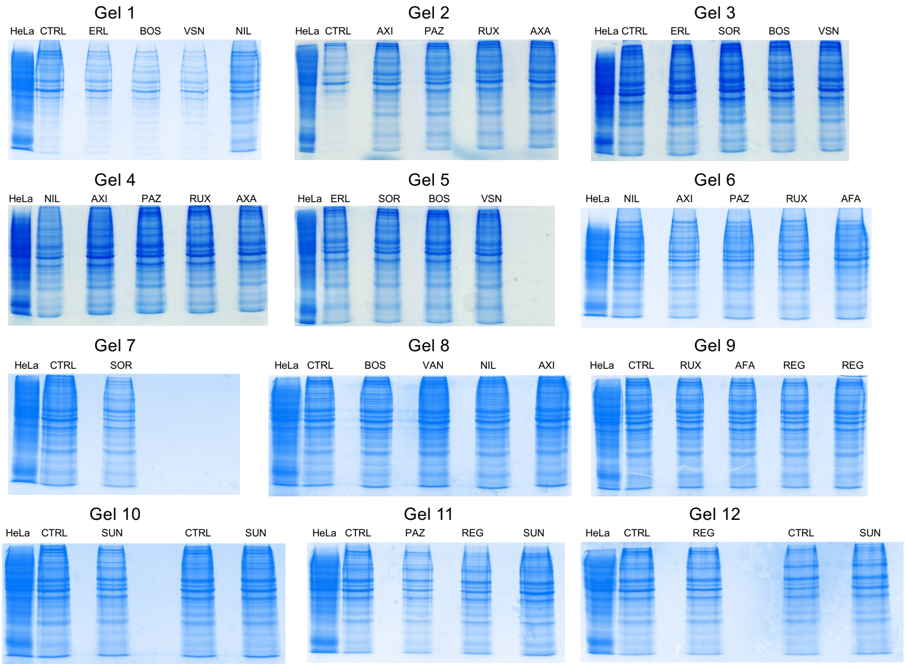

Supplementary Figure 1. Examples of protein quantification via serial SDS-PAGE. Twenty five micrograms of internally-prepared HeLa cell protein was loaded along with the proteins recovered from the KI treated PMC-D line and separated on 10% SDS-PAGE gels. The gel was stained with Coomassie brilliant blue (CBB) and the staining intensities were measured. The protein CBB densities in drug-treated samples were compared to those of the HeLa cells for the same gel, and the protein amounts in each sample were then estimated. Example protein density measurements for the gels shown here are reported in **Online-only Table 1**.

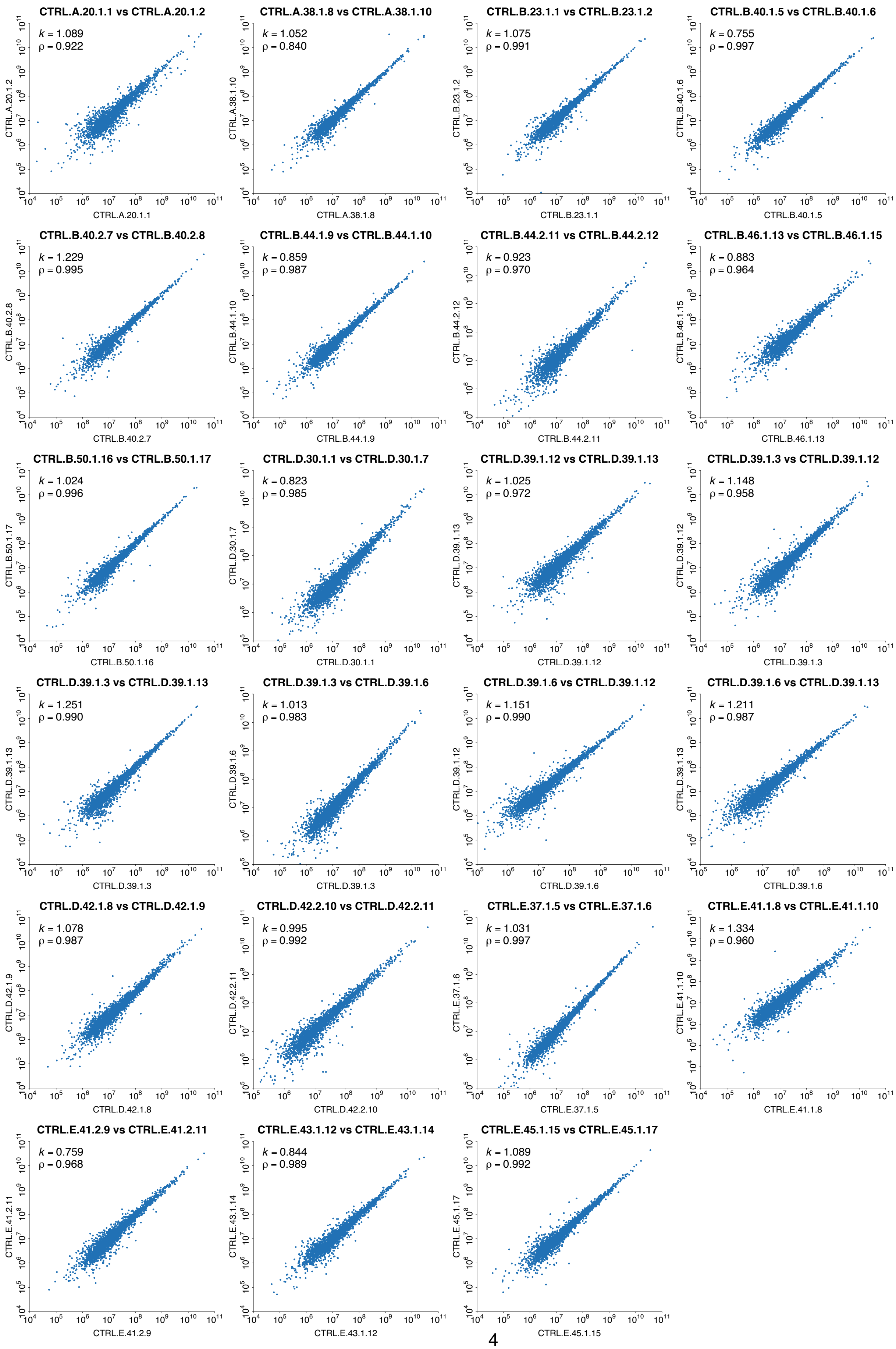
CTRL.A.20.1.1 vs CTRL.A.20.1.2
k = 1.089
ρ = 0.922
CTRL.A.38.1.8 vs CTRL.A.38.1.10
k = 1.052
ρ = 0.840
CTRL.B.23.1.1 vs CTRL.B.23.1.2
k = 1.075
ρ = 0.991
CTRL.B.40.1.5 vs CTRL.B.40.1.6
k = 0.755
ρ = 0.997
CTRL.B.40.2.7 vs CTRL.B.40.2.8
k = 1.229
ρ = 0.995
CTRL.B.44.1.9 vs CTRL.B.44.1.10
k = 0.859
ρ = 0.987
CTRL.B.44.2.11 vs CTRL.B.44.2.12
k = 0.923
ρ = 0.970
CTRL.B.46.1.13 vs CTRL.B.46.1.15
k = 0.883
ρ = 0.964
CTRL.B.50.1.16 vs CTRL.B.50.1.17
k = 1.024
ρ = 0.996
CTRL.D.30.1.1 vs CTRL.D.30.1.7
k = 0.823
ρ = 0.985
CTRL.D.39.1.12 vs CTRL.D.39.1.13
k = 1.025
ρ = 0.972
CTRL.D.39.1.3 vs CTRL.D.39.1.12
k = 1.148
ρ = 0.958
CTRL.D.39.1.3 vs CTRL.D.39.1.13
k = 1.251
ρ = 0.990
CTRL.D.39.1.3 vs CTRL.D.39.1.6
k = 1.013
ρ = 0.983
CTRL.D.39.1.6 vs CTRL.D.39.1.12
k = 1.151
ρ = 0.990
CTRL.D.39.1.6 vs CTRL.D.39.1.13
k = 1.211
ρ = 0.987
CTRL.D.42.1.8 vs CTRL.D.42.1.9
k = 1.078
ρ = 0.987
CTRL.D.42.2.10 vs CTRL.D.42.2.11
k = 0.995
ρ = 0.992
CTRL.E.37.1.5 vs CTRL.E.37.1.6
k = 1.031
ρ = 0.997
CTRL.E.41.1.8 vs CTRL.E.41.1.10
k = 1.334
ρ = 0.960
CTRL.E.41.2.9 vs CTRL.E.41.2.11
k = 0.759
ρ = 0.968
CTRL.E.43.1.12 vs CTRL.E.43.1.14
k = 0.844
ρ = 0.989
CTRL.E.45.1.15 vs CTRL.E.45.1.17
k = 1.089
ρ = 0.992

Supplementary Figure 2. Reproducibility of proteomic quantification for technical replicates. Comparison of normalized MS1 LFQ intensities for two technical replicates of the same biological sample that was run on a different SDS-PAGE gel and processed for MS/MS analysis on a different day. Pearson correlations show strong agreement between multiple runs as both coefficients, ρ, and slopes, *k*, are at or near unity.

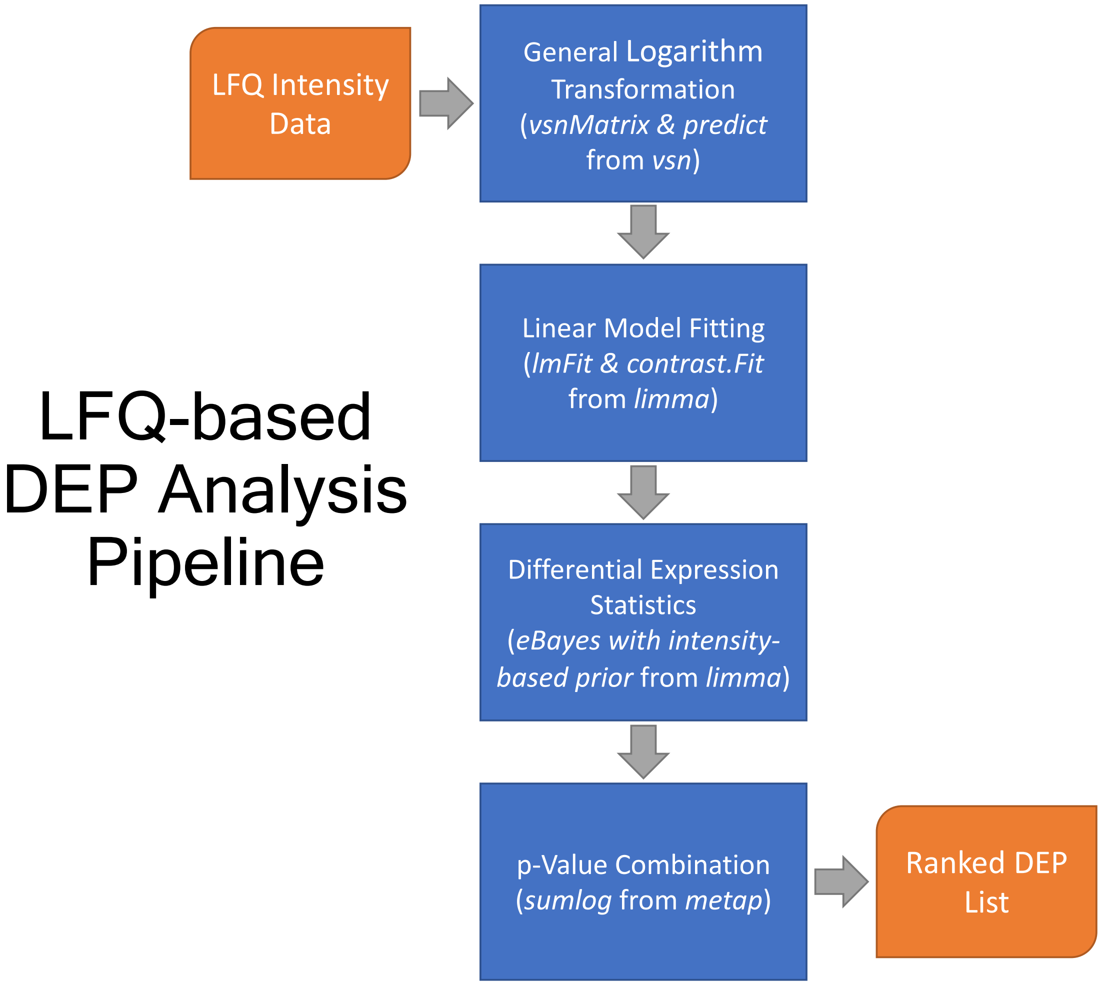

Supplementary Figure 3. Workflow for identifying differentially expressed proteins from LFQ intensities.

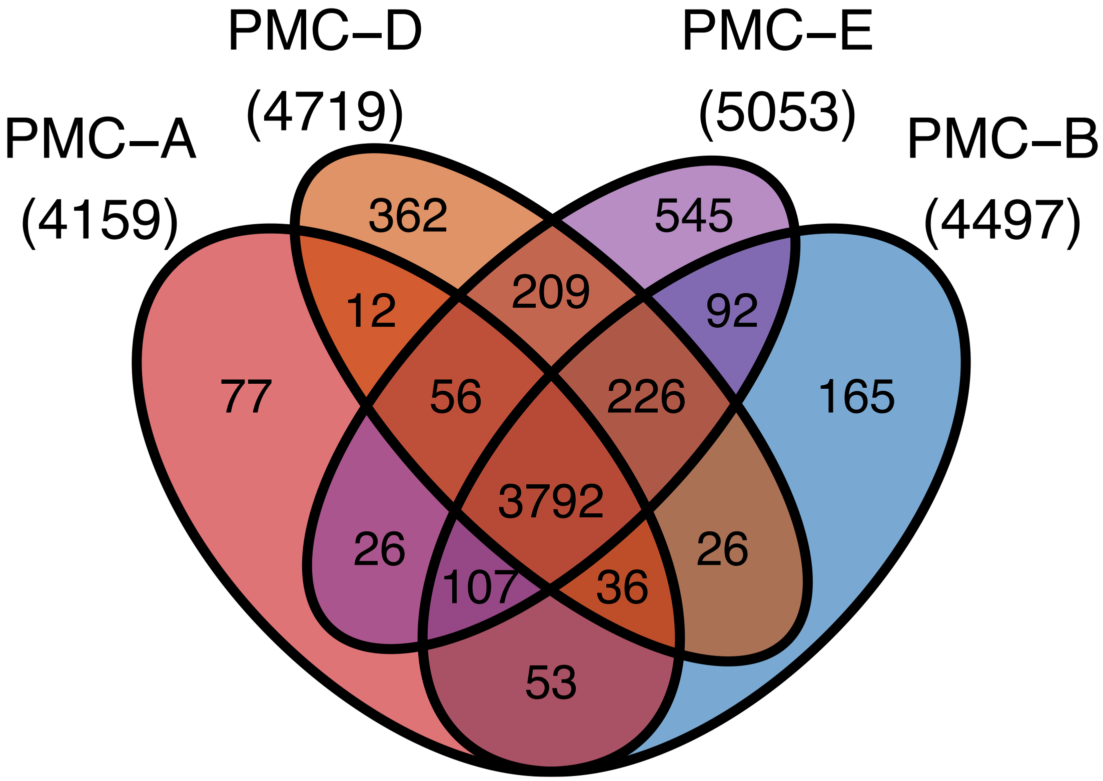

Supplementary Figure 4. Coverage of identified proteins that map onto genes. Venn diagram of the overlapping and unique proteins across all samples for the four human cardiomyocyte-like cell lines (PMC-A, B, D, E) that map onto genes.

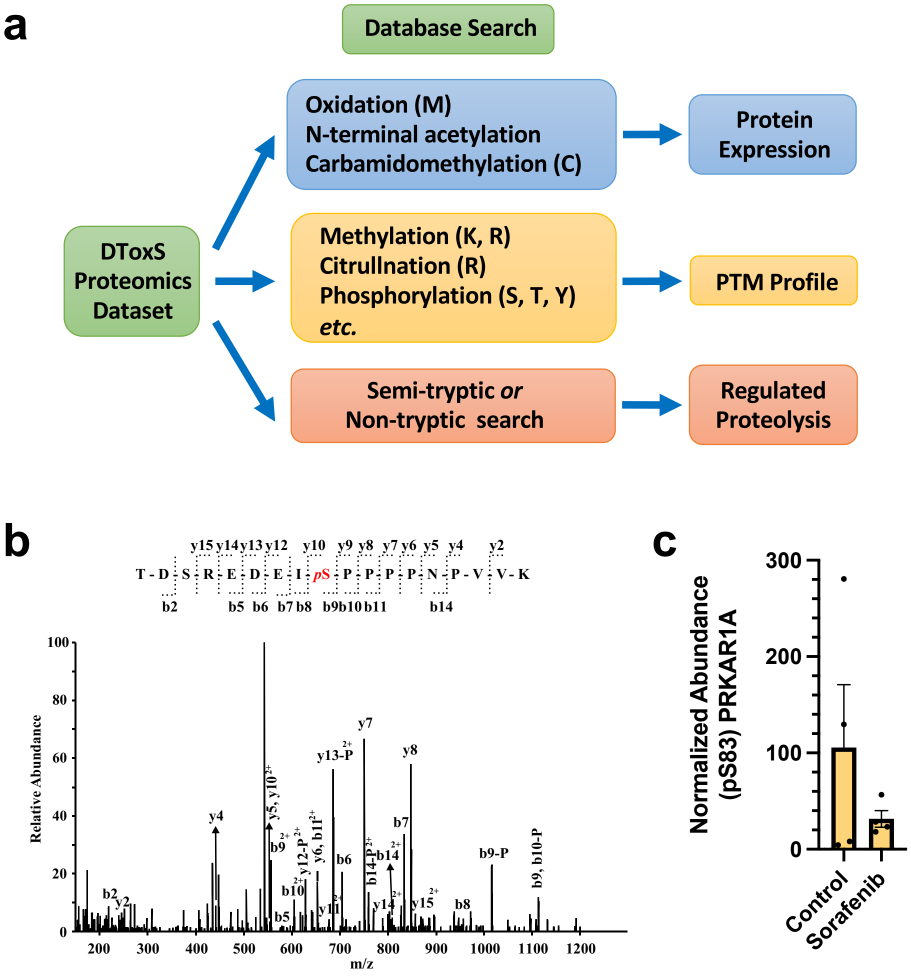

Supplementary Figure 5. Using the complementary proteomics search methods to identify drug-regulated changes of PTMs. (a) The DToxS proteomics dataset can be analyzed with different protein database search methods to obtain different pertinent information, including the changes of protein expression, PTMs and regulated proteolysis of specific peptides. **(b)** Example of a phosphorylation site observed in cardiomyocyte-like human cell line PMC-B treated with multi-kinase inhibitor sorafenib. **(c)** Normalized abundance of the phosphorylated S83 residue for the cAMP dependent kinase A regulatory subunit (PRKAR1A), well known for its functional role in numerous cardiac processes, is significantly reduced in sorafenib treated cells.

Supplementary Table 1. Sample sizes for the KI treated groups per cell line

Drug Treatment	PMC-A	PMC-B	PMC-D	PMC-E
AFA	4	4	4	5
AXI	4	4	4	5
BOS	4	4	4	5
CAB	0	4	0	0
DAB	0	4	0	0
DAS	0	4	0	0
ERL	2	4	4	2
GEF	0	4	4	5
IMA	0	0	0	3
LAP	0	1	0	3
NIL	4	4	4	5
PAZ	4	4	4	5
PON	0	4	0	0
REG	4	4	4	5
RUX	4	4	4	5
SOR	3	3	0	4
SUN	3	4	4	6
TOF	0	4	4	4
TRA	0	4	0	0
TRS	0	4	0	4
VAN	4	3	4	5
VEM	0	3	0	0

* Four human cardiomyocyte-like cell lines; 35 separate experiments; 24 drugs
** Control (CTRL) samples: 13 biological replicates for PMC-A, 23 biological replicates for PMC-B, 13 biological replicates for PMC-D, and 22 biological replicates for PMC-E.
